# Regression of the Flow Signal from the Neovascular Network in AMD Neovascular Membranes Treated with Faricimab

**DOI:** 10.3390/diagnostics14232653

**Published:** 2024-11-25

**Authors:** Maria Cristina Savastano, Emanuele Crincoli, Lisa Toto, Maria Oliva Grassi, Flavia Chiosi, Alfonso Savastano, Clara Rizzo, Rodolfo Mastropasqua, Francesco Boscia, Stanislao Rizzo

**Affiliations:** 1 Ophthalmology Unit, “Fondazione Policlinico Universitario A. Gemelli IRCCS”, 00168 Rome, Italy; mariacristina.savastano@gmail.com (M.C.S.); asavastano21@gmail.com (A.S.); stanislao.rizzo@gmail.com (S.R.); 2 Department of Head and Neck Medicine, Catholic University of “Sacro Cuore”, 00168 Rome, Italy; 3 Ophthalmology Clinic, Department of Medicine and Science of Ageing, University G. D’Annunzio Chieti-Pescara, 66100 Chieti, Italy; l.toto@unich.it (L.T.); rodolfo.mastropasqua@unich.it (R.M.); 4 Department of Translational Biomedicine Neuroscience, University of Bari “Aldo Moro”, 70121 Bari, Italy; mariaolivagrassi@gmail.com (M.O.G.); francesco.boscia@uniba.it (F.B.); 5 Department of Ophthalmology, Azienda Ospedaliera dei Colli-Ospedale Monaldi, 80131 Naples, Italy; flaviachiosi@yahoo.it; 6 Ophthalmic Unit, Department of Neurosciences, Biomedicine and Movement Sciences, University of Verona, 37129 Verona, Italy; clararizzo2@gmail.com; 7 Consiglio Nazionale delle Ricerche (CNR), Istituto di Neuroscienze, 56124 Pisa, Italy

**Keywords:** faricimab, anti-VEGF, age-related macular degeneration, OCTA, regression, neovascularization

## Abstract

Objectives: To report the occurrence of the regression of the flow signal from the neovascular network in macular neovascularizations (MNVs), developing in the context of age-related macular degeneration (AMD), treated with faricimab in a treat-and-extend regimen. Methods: Eyes affected by AMD-related MNV and treated with faricimab intravitreal injections in a treat-and-extend (TE) regimen were consecutively retrospectively screened in five specialized retina centers. Changes in neovascular network characteristics during the course of the treatment were analyzed. The availability of high-quality optical coherence tomography angiography (OCTA) at the beginning of the treatment and at the regression of the MNV was necessary for inclusion. According to greatest linear diameter (GLD) changes during treatment, eyes were divided into three groups: a complete regression (CR) group, a partial remission (PR) group (a reduction of at least 50% of the GLD from baseline to last follow-up), and a stable group (stable/showing a reduction lower than 50% of the GLD from baseline to follow up). Results: One hundred and ten (110) eyes were included. The CR group was composed of 12 eyes (10.9%), while the PR group represented 60.9% of the study population. CR occurred after a mean of 6.0 ± 1.4 months, ranging from 4 to 8 months. Time to regression was significantly lower in eyes naïve to treatment before the study compared with the others (*p* = 0.022). A significantly lower baseline GLD was detected in the CR group (1292.2 ± 195.6 μm) compared with the PR group (1324.6 ± 135.6 μm) and the stable group (1412.5 ± 110.9 μm) (omnibus *p* = 0.003). Conclusions: Complete regression of the flow signal from the MNV neovascular network documented with OCTA may occur during TE regimens with faricimab. In treatment-naïve eyes, regression occurs earlier during the treatment.

## 1. Introduction

Age-related macular degeneration (AMD) is a multifactorial, progressive, chronic disease of the retina that affects 170 million people worldwide, with an estimated prevalence that will reach 288 million by 2040 [1]. While neovascular AMD (nAMD) represents only 10–15% of AMD eyes, it is responsible for around 80% of cases of severe visual loss attributable to retinal exudation, hemorrhage, and disciform scarring [2]. Since the approval of anti-vascular endothelial growth factor (VEGF) pharmacotherapy in 2006, the impact on the visual prognosis of AMD has been considerably reduced, removing neovascular AMD from the list of incurable diseases [3]. Nevertheless, although anti-VEGF treatment was able to slow or halt retinal degeneration in the majority of cases, it still required a high treatment burden, and the final visual prognosis was often unsatisfactory. This led researchers to persist in their efforts to find better therapeutic solutions [4]. Among them, a novel bispecific monoclonal antibody that targets both VEGF-A and angiopoietin-2 (Ang-2) receptors, faricimab, has been recently released onto the market. Due to the important role played by the Ang-2 receptor (Tie-2) in vascular stability, local control of inflammation, and angiogenesis, the simultaneous inhibition of both pathways was expected to produce a stronger inhibitory effect on the growth and survival of macular neovascularizations (MNVs) as well as exudation [5]. Consistent with expectations, positive outcomes in faricimab phase II trials led to the initiation of the phase III studies TENAYA and LUCERNE, which confirmed non-inferiority to aflibercept and attested to the surprisingly durable effect of the treatment [6,7,8,9]. To the present date, the release onto the market of faricimab allows researchers to test the efficacy of the treatment in real-life settings. The multiple-target approach is also expected to have positive effects on the anatomy of the neovascular network, potentially inducing the regression of MNV. In fact, the onset of neovascularization is made possible by two different processes, namely angiogenesis and arteriogenesis. Angiogenesis is the sprouting of new capillaries, while arteriogenesis is the dilation of pre-existing channels by the active proliferation and remodeling of the vessel wall in response to high flow states [10,11]. An intriguing difference is that angiogenesis is highly VEGF-dependent, while arteriogenesis is not VEGF-dependent [12]. For example, the integrity of the innate and adaptive immune systems is needed for arteriogenesis to be fully accomplished, which is consistent with the report of MNV regression as a consequence of systemic treatment with infliximab [13,14]. Similarly, other ligands are required for the ultimate completion of the process of arteriogenesis, including Ang-2 [15]. In this context, we aim to report and describe the regression of the flow signal within the MNV neovascular network, as visualized with optical coherence tomography angiography (OCTA) in nAMD eyes as a result of faricimab injections performed in a treat-and-extend (TE) regimen.

## 2. Materials and Methods

The study was conducted in 5 specialized retina centers located in Europe (the Ophthalmology Unit at Fondazione Policlinico Universitario Agostino Gemelli, IRCCS in Rome, Italy; Ophthalmology Clinic, Department of Medicine and Science of Ageing, University G. D’Annunzio Chieti-Pescara in Italy; the Department of Translational Biomedicine Neuroscience, University of Bari “Aldo Moro” in Bari, Italy; the Department of Ophthalmology, Azienda Ospedaliera dei Colli-Ospedale Monaldi in Naples, Italy; and Centre Ophtalmologique Paris Gare du Nord in Paris, France). The study was approved by the Institutional Ethics Committee of Fondazione Policlinico Universitario Agostino Gemelli, IRCCS Università Cattolica del Sacro Cuore (approval code: NCT05747144, approval date: 15 January 2021). Each patient was individually approached and asked for permission for inclusion in the study. Under these circumstances, informed consent was collected and a complete explanation of the target protocol was fully provided, in conformity with the declaration of Helsinki.

Eyes diagnosed with MNV related to nAMD and treated with faricimab intravitreal injections (IVIs) in a TE regimen were consecutively retrospectively screened from March 2023 to February 2024. AMD was defined as the presence of large macular drusen (>63 μm) and RPE abnormalities with pigmentary changes in the macular region documented with both the slit lamp examination and OCT B-scan acquisitions [16]. The presence of an MNV was assessed at baseline with both OCT B-scan and OCTA. Fluorescein angiography (FA) was performed only in case of doubt. On OCTA, MNV was identified as an abnormal OCTA signal increase in the outer retina to the choriocapillaris complex (ORCC) and/or in the outer avascular retina. On FA, MNV was detected as a hyperfluorescent lesion characterized by leakage and staining located in the exudation area, with or without blocking from a possible retinal hemorrhage. The exclusion criteria were the presence of overlapping retinal diseases, a history of uveitis, previous ocular trauma or systemic diseases influencing OCTA, and proangiogenic microenvironment (i.e., diabetes, glaucoma, and optic nerve diseases). The availability of high-quality (Optovue Q and Solix Fullrange 6/10; Heidelberg Q > 15; Topcon Q 7/10) OCT B-scans and OCTA acquisitions at the beginning of the treatment, at remission of exudation, and at the regression of the MNV was necessary for inclusion. Acquisitions that did not allow a complete 360° view of the MNVs’ margins were not considered for the study. OCT B-scan and OCTA images were acquired using Solix FullRange OCT/OCTA (Optovue Inc., Freemont, CA, USA—version 2019 V1.0.0.305), Spectralis HRA OCT software version 5.4.7.0 (Heidelberg Engineering, Inc., Heidelberg, Germany), or Topcon ImageNet 6 (DRI OCT Triton, Topcon Corporation, Tokyo, Japan). The protocol for Solix FullRange was a horizontal linear B-scan and 6.4 × 6.4 mm angioscan pattern centered on the fovea. The protocol for Spectralis HRA OCT software version 5.4.7.0 was a horizontal linear raster composed of 9 averaged OCT B-scans (1024 A-scans per line) at a 60 µm interval, covering an area of 20° × 20°. OCTA acquisition consisted of 10° × 10° volumes centered on the capillary-free zone (CFZ) (foveal avascular zone). Images were acquired using 85,000 A-scans per second using a light source centered at a wavelength of either 840 nm (SD-OCTA), achieving an optical axial resolution of 3.9 µm and a transverse resolution of 5.7 µm. As concerns Topcon ImageNet 6, the scanning area was a 4.5 × 4.5 mm horizontal B-scan raster, and the angiogram was centered on the macula. MNVs encountering OCTA regression after at least 4 months of treatment (the duration of loading as per trial protocols) were considered complete regressions. A 4-week-step TE regimen was followed after the induction of four monthly intravitreal injections. Best corrected visual acuity (BCVA) assessments with a Snellen chart and a slit lamp examination were also performed at baseline, at remission of exudation, and at the regression of the flow signal. Remission of the exudation was defined as the disappearance of all signs of exudation during the course of the treatment, including intraretinal and/orsubretinal fluid and/or subretinal hemorrhage.

### 2.1. Outcome Measures

Structural changes occurring in the MNV during treatment were evaluated using several parameters, including MNV greatest linear diameter (GLD), MNV height, the morphology of the MNV, and MNV vessel density (VD). MNV GLD was defined as the maximum linear distance measured between opposite margins of the MNV, and it was manually assessed on enface OCTA by two expert graders. As the first step, enface segmentation was manually refined to focus on the section allowing the widest visibility of the MNV network. Second, non-binarized OCTA enface acquisitions were processed for background noise artifact removal using ImageJ software v.2.14.0 (NIH, Bethesda, MD, USA). GLD was then independently measured by the graders, and the mean of the 2 measurements for each acquisition was considered the final GLD value. Last, references to segmentation were registered and applied to follow-up acquisitions in order to allow the evaluation of changes occurring in the originally chosen section of the MNV. According to GLD changes during treatment, eyes were divided into 3 groups: a complete regression (CR) group, a partial remission (PR) group (reduction of at least 50% of the GLD from baseline to last follow-up), and a stable group (stable/showing a reduction lower than 50% of the GLD from baseline to follow-up). CR was defined as the disappearance of the OCTA signal from all available sections scanning the MNV area. The mean height of the MNV was assessed on the fovea-crossing OCT B-scan line available for each timepoint and was calculated considering the mean linear distance from the inner margin of the MNV to the outer margin of the MNV. The inner margin was delimited by the outer margin of the RPE located above the pigment epithelial detachment (PED) in cases of type I MNV and by the inner surface of the MNV lesion in cases of type II and mixed MNV. The outer margin was delimited by the Bruch membrane. Mean linear distance was calculated on the basis of the segmented area using integral calculus. The morphology of the MNV at enface angiogram was evaluated, dividing the MNVs into tree-like patterns, glomerular patterns, and fragmented patterns [17]. MNVs were also qualitatively identified as immature, mature, or hypermature according to Xu et al.’s [18] description. Lastly, VD was calculated using binarized and segmented OCTA angiograms as the percentage area of white pixels (vascular area) in the total segmented area [19,20].

### 2.2. Statistical Analysis

Statistical analysis was performed using SPSS v.26 (IBM statistics, IBM Corp., Armonk, NY, USA). Quantitative variables were normally distributed according to the Shapiro–Wilk test when considering the total population and were thus expressed using the mean and standard deviation. The comparison of quantitative variables between CR eyes, the PR group, and the stable group was performed with ANOVA for independent samples. The analysis of the correlation between GLD at baseline and time to regression as well as the analysis of the correlation between MNV height at baseline and time to regression were performed using Pearson’s correlation. Qualitative variables were expressed as the number of cases out of the total and the percentage. Comparison of the 3 groups as concerns qualitative variables was performed using the Chi-square test or Fisher’s exact test, as appropriate (less than 5 cases for at least one category). Post-hoc analysis was performed using the Tukey method for ANOVA and the Bonferroni method for the Chi-square test. A *p*-value of <0.05 was considered statistically significant.

## 3. Results

**Figure 1:**
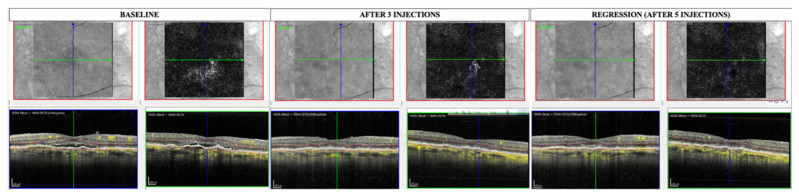
Enface and B-scan OCTA at baseline, at remission of exudation, and at the regression of the MNV are shown. MNV = macular neovascularization; OCTA = optical coherence tomography angiography. Images were acquired with Spectralis HRA OCT software version 5.4.7.0 (Heidelberg Engineering, Inc., Heidelberg, Germany).

**Figure 2:**
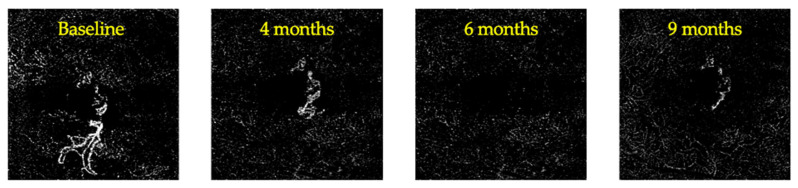
MNV showing regression and subsequent reappearance. MNV = macular neovascularization.

**Figure 3:**
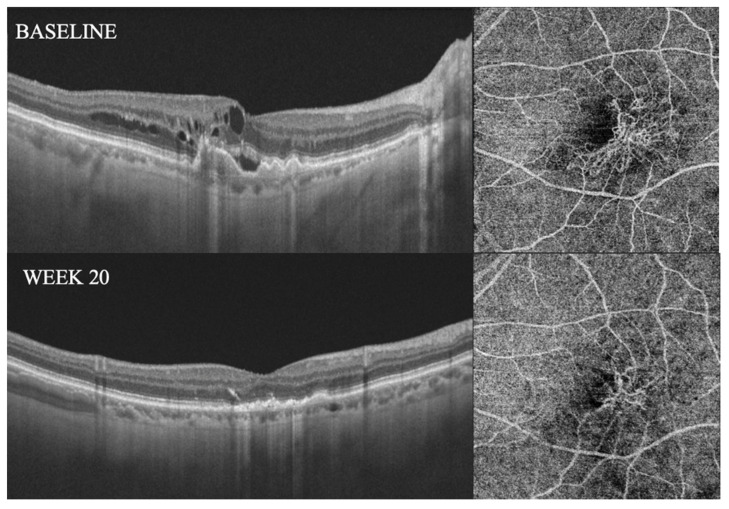
Decrease in GLD from baseline to last follow-up in an eye that did not show complete regression of the MNV. GLD = greatest linear diameter; MNV = macular neovascularization.

One hundred and ten (110) eyes met the eligibility criteria and were thus included. Twelve (12) eyes of twelve patients (12/110, 10.9%) showed complete regression of the MNV during follow-up (CR group). In this group, remission of the exudation occurred after 3.38 ± 1.32 months (ranging from 2 to 6 months), and complete regression of the MNV occurred after 6.0 ± 1.4 months, ranging from 4 to 8 months (see Figure 1). A reappearance of the MNV was detected in 3 of the 12 cases, with a minimum interval of 12 weeks from regression (see Figure 2). Table 1 provides a description of demographic data as well as imaging and clinical course information for each case from the CR group. A decrease in GLD of >50% at the end of follow-up (PR group) was experienced in 67/110 patients (60.9%) in the study population, with a mean time to remission of the exudation of 4.62 ± 1.59 months (ranging from 2 to 8 months) (see Figure 3). No significant differences in terms of age, sex, or follow-up duration were noted between the CR group, PR group, and stable group (see Table 2).

In the CR group, 6/12 (50.0%) of eyes were naïve to anti-VEGF treatment, which was significantly higher than the prevalence detected in the PR group (omnibus *p* = 0.029, see Table 2; post-hoc CR group vs. PR group *p* = 0.009; post-hoc CR group vs. stable group *p* = 0.196; post-hoc PR group vs. stable group *p* = 0.149). Ten out of twelve (83.3%) MNVs in the CR group were type I, while the rest (2/10, 16.7%) were type II. A similar prevalence of type I MNVs was detected in the PR group (86.6%) and the stable group (80.6%) (*p* = 0.675). A glomerular pattern was found in 5/12 (41.6%) MNVs, while tree-like patterns and fragmented patterns were found in 4/12 (33.3%) and 3/12 (25.0%) MNVs, respectively. No significant differences in terms of MNV pattern were detected compared to non-regressing eyes (*p* = 0.744). Among eyes encountering regression, 5/12 (41.7%) were immature MNVs, 5/12 (41.7%) were mature MNVs, and 2/12 were hypermature MNVs (16.6%). A higher prevalence of hypermature MNVs was detected in both the stable group (12/31, 38.7%) and the PR group (20/67, 29.9%), but this difference did not reach statistical significance (*p* = 0.490). The mean height of the MNV at baseline in the CR group was 1114.9 ± 33.3 μm, which was not significantly different from the one measured in the two remaining study groups (*p* = 0.784). By contrast, the mean GLD at baseline was significantly lower in the CR group (1292.2 ± 195.6 μm) compared with the PR group (1324.6 ± 135.6 μm) and the stable group (1412.5 ± 110.9 μm) (omnibus *p* = 0.003; post-hoc CR group vs. PR group *p* = 0.047; post-hoc CR group vs. stable group *p* = 0.006; post-hoc PR group vs. stable group *p* = 0.039).

No significant differences in VD were detected at baseline between the three study groups (*p* = 0.592). In the CR group, the time to regression of the MNV was significantly lower in naïve eyes than in previously treated eyes (*p* = 0.022). Also, time to regression showed a borderline statistically significant correlation with GLD at baseline (*p* = 0.08, r = 0.54, r squared = 0.29). No significant correlation between MNV height or time to regression of the MNV was detected (*p* = 0.601).

## 4. Discussion

OCTA has improved our ability to non-invasively detect and characterize the neovascular network of MNVs, also allowing us to analyze its response to treatment [21,22,23].

We know from the literature that Tie-2 is prevalently located on big vessels in pathologic neovascularizations and that Ang-2 is prevalently expressed in core neovessels that are responsible for resprouting of neovascularizations [24]. Therefore, the inhibition of this pathway can potentially result in a strong inhibitory effect at the level of the large trunks and core structures of the MNV, potentially leading to the regression of the neovascular network. In our cohort, we report regression of the flow signal in around 10% of MNVs treated with faricimab, with a mean time to regression of 6 months. Moreover, despite not encountering complete regression, the remaining 60% of the study population experienced a reduction in the MNV GLD of at least 50% of the baseline value. As concerns the adverse effects of treatment in our study population, one case of hypertensive uveitis was diagnosed after the fourth injection, which is compatible with other reports in the literature [25,26].

Interestingly, the prevalence of treatment-naïve eyes was significantly higher in the group showing complete regression compared with the rest of the population, suggesting that this subgroup might be more sensitive to the inhibitory effect elicited by the treatment. Moreover, in treatment-naïve eyes, complete regression occurred significantly earlier during the treatment compared with eyes previously treated with other anti-VEGF agents. By contrast, despite a higher prevalence of hypermature MNVs in the stable group, no significant differences in MNV morphology or pattern were detected between the study groups. However, the lack of statistical significance could be explained by the limited sample size, especially concerning the number of cases showing complete regression.

By contrast, while demographic and morphological factors like sex, age, and baseline MNV height showed no significant correlation with the time to regression of the MNV, a borderline correlation was observed for GLD at baseline. This is consistent with the fact that eyes experiencing complete regression of the MNV showed a significantly lower GLD at baseline compared with the rest of the population, meaning that smaller MNVs are more likely to encounter an involution during treatment. Other authors have described different regression phenomena, such as that of the transformation of type II MNVs into type I MNVs during the course of treatment with anti-VEGF injections [27]. This modification was considered a form of regression from an anatomical and a prognostic standpoint. In fact, not only does the transition from a type I to a type II MNV imply MNV envelopment by the RPE (anatomical regression), but it also translates into a positive effect on prognosis considering the lower rate of subretinal fibrosis and the overall final visual acuity reported in this type of MNV. In our cohort, a similar prevalence of type I and type II MNVs was detected in the three study groups, and no transition from type II to type I was reported as a result of the treatment. Also, other authors have described the involution of the PED containing the MNV as a signal of the conversion toward an atrophic phenotype after anti-VEGF treatment [28]. As some authors describe perimacular sensitivity to be a prognostic factor for response to treatment in nAMD, we encourage further studies to investigate the association between microperimetry results and the regression of the neovascular network in eyes treated with faricimab injections [29,30].

In the specific case of eyes treated with faricimab, the relatively high incidence of regression may be explained by the effect of Ang-2 receptor inhibition on large-caliber vessels. In fact, the inhibition of this pathway could result in a strong inhibition of angiogenesis targeting not only small-caliber vessels but also large-caliber ones [5]. Interestingly, 25% of patients in the CR group experienced a reappearance of MNV after a minimum interval of 12 weeks from regression. A similar occurrence was reported in the literature for eyes treated with ranibizumab with a shorter average interval of 2 weeks. This event could be explained by a decrease in blood flow within the neovascular network below the threshold of detection of the device during the first weeks after treatment. The longer duration of this temporary flow signal regression in eyes treated with faricimab is compatible with the longer duration of the effect of treatment, as demonstrated by the longer time to exudation reported in the literature. Likewise, the disappearance of the OCTA signal in the remaining 75% of participants in the CR group could be the result of a reduction in blood flow within the membrane below the detection threshold of the OCTA device. For this reason, among the limitations of our study, we can mention the lack of confirmation of the regression of the neovascular network using either ICGA or anatomopathological samples. Nevertheless, subthreshold perfusion of the MNV could still be considered a valid anatomical outcome of anti-VEGF treatment, indicating a state of inactivity of the MNV. Certainly, the analysis of the modifications occurring in these inactivated MNVs performed in a longer follow-up setting would be of scientific interest and will be the object of future investigations. Lastly, the lack of a control group and the use of a spectral domain technology for OCTA imaging should be regarded as limitations. In fact, swept source imaging is more accurate in the evaluation of cases with a disturbed signal (such as in the presence of perilesional halo due to exudation) and in the assessment of quantitative OCTA parameters [31,32].

Nonetheless, we believe that the relatively high prevalence of the complete regression of the OCTA signal reported with faricimab treatment in the present study is a finding that deserves scientific attention. In fact, to the best of our knowledge, this is the first report in the literature describing the regression of the MNV during a TE regimen with faricimab. If further confirmed, it could pave the way for new objectives in MNV treatment, establishing the disappearance of the neovascular network as the ultimate therapeutic target.

## Figures and Tables

**Table 1:** Detailed report of characteristics of MNVs encountering regression after treatment with intravitreal faricimab. F = female; GLD = greatest linear diameter; M = male; MNV = macular neovascularization.

	Sex	Treatment-Naïve	Type of MNV	Type of Neovascular Network	MNV Height at Baseline (μm)	MNV GLD at Baseline (μm)	Time to Remission of Exudation (Months)	Time to Regression of the MNV (Months)
1	M	Yes	I	Glomerular Immature	105.1	1223	2	4
2	F	No	I	Tree-like Mature	167.7	1127	3	4
3	M	Yes	I	Fragmented Mature	98.0	1608	4	6
4	M	Yes	I	Fragmented Hypermature	124.8	1098	1	4
5	F	Yes	I	Glomerular Immature	73.2	1455	3	6
6	M	No	I	Tree-like Mature	68.2	1397	4	6
7	F	No	I	Fragmented Hypermature	178.4	1290	5	8
8	M	No	II	Tree-like Mature	136.3	1505	6	8
9	F	Yes	I	Glomerular Immature	109.1	1560	5	8
10	F	No	II	Tree-like Immature	129.3	1081	4	6
11	F	Yes	I	Glomerular Immature	77.5	1107	3	6
12	M	No	I	Glomerular Mature	112	1055	3	6

**Table 2:** Comparison of the characteristics of the three study groups. CR = complete regression; GLD = greatest linear diameter; MNV = macular neovascularization; PR = partial regression; VD = vessel density. * = statistically significant value.

	CR Group (12/110)	PR Group (67/110)	Stable Group (31/110)	*p*
Age	71.3 ± 6.9	70.1 ± 7.2	70.9 ± 7.0	0.996
Sex	6/12 (50.0%)	32/67 (47.7%)	14/31 (45.2%)	0.952
Follow-up (months)	7.9 ± 1.2	8.2 ± 1.4	8.0 ± 1.3	0.865
Naïve eyes	6/12 (50.0%)	11/67 (16.4%)	9/31 (29.1%)	0.029 *
MNV type	I = 10/12 (83.3%) II = 2/12 (16.7%)	I = 58/67 (86.6%) II = 9/67 (13.4%)	I = 25/31 (80.6%) II = 6/31 (19.3%)	0.675
MNV pattern	Glomerular = 5/12 (41.6%) Tree-like = 4/12 (33.3%) Fragmented = 3/12 (25.0%)	Glomerular = 23/67 (34.3%) Tree-like = 24/67 (35.8%) Fragmented = 20/67 (29.8%)	Glomerular = 8/31 (25.8%) Tree-like = 10/31 (32.3%) Fragmented = 13/31 (41.9%)	0.744
MNV morphology	Immature = 5/12 (41.6%) Mature = 5/12 (41.6%) Hypermature = 2/12 (16.7%)	Immature = 25/67 (37.3%) Mature = 22/67 (32.8%) Hypermature = 20/67 (29.9%)	Immature = 7/31 (22.6%) Mature = 12/31 (38.7%) Hypermature = 12/31 (38.7%)	0.490
MNV height at baseline (μm)	1114.9 ± 33.3	1190.8 ± 42.0	1074.9 ± 35.6	0.784
GLD at baseline (μm)	1292.2 ± 195.6	1324.6 ± 135.6	1412.5 ± 110.9	0.003 *
VD at baseline (%)	40.34 ± 6.1	40.92 ± 5.8	39.93 ± 5.7	0.592

## Data Availability

The data presented in this study are available upon request from the corresponding author due to privacy.
